# Community Event-Based Surveillance for Ebola Virus Disease in Sierra Leone: Implementation of a National-Level System During a Crisis

**DOI:** 10.1371/currents.outbreaks.d119c71125b5cce312b9700d744c56d8

**Published:** 2016-12-07

**Authors:** Erin Stone, Laura Miller, Joseph Jasperse, Grayson Privette, Juan Cruz Diez Beltran, Amara Jambai, John Kpaleyea, Alfred Makavore, Mohamed Foday Kamara, Ruwan Ratnayake

**Affiliations:** International Rescue Committee, Freetown, Sierra Leone; International Rescue Committee, Freetown, Sierra Leone; International Rescue Committee; International Rescue Committee, Freetown, Sierra Leone; Save the Children, Freetown, Sierra Leone; Ministry of Heath and Sanitation, Freetown, Sierra Leone; International Rescue Committee, Freetown, Sierra Leone; CARE International, Freetown, Sierra Leone; ABC Development, Sierra Leone; International Rescue Committee, New York, New York, USA

**Keywords:** Case Study, Community-based, Ebola Virus Disease, Event triggers, Surveillance

## Abstract

INTRODUCTION: There are few documented examples of community networks that have used unstructured information to support surveillance during a health emergency. In January 2015, the Ebola Response Consortium rapidly implemented community event-based surveillance for Ebola virus disease at a national scale in Sierra Leone.

METHODS: Community event based surveillance uses community health monitors in each community to provide an early warning system of events that are suggestive of Ebola virus disease transmission. The Ebola Response Consortium, a consortium of 15 nongovernmental organizations, applied a standardized procedure to implement community event-based surveillance across nine of the 14 districts. To evaluate system performance during the first six months of operation (March to August 2015), we conducted a process evaluation. We analyzed the production of alerts, conducted interviews with surveillance stakeholders and performed rapid evaluations of community health monitors to assess their knowledge and reported challenges.

RESULTS: The training and procurement of supplies was expected to begin in January 2015 and attain full scale by March 2015. We found several logistical challenges that delayed full implementation until June 2015 when the epidemic was past its peak. Community health monitors reported 9,131 alerts during this period. On average, 82% of community health monitors reported to their supervisor at least once per week. Most alerts (87%) reported by community health monitors were deaths unrelated to Ebola. During the rapid evaluations, the mean recall by community health monitors was three of the six trigger events. Implementation of the national system achieved scale, but three months later than anticipated.

DISCUSSION: Community event based surveillance generated consistent surveillance information during periods of no- to low-levels of transmission across districts. We interpret this to mean that community health monitors are an effective tool for generating useful, unstructured information at the village level. However, to maximize validity, the triggers require more training, may be too many in number, and need increased relevance to the context of the tail end of the epidemic.

## Introduction

In August 2014, the WHO declared the outbreak of Ebola virus disease (EVD) in West Africa to be a Public Health Emergency of International Concern.^1^ The number of EVD cases across the region rose sharply during the following four months, with Sierra Leone having reported a total of 7,476 confirmed EVD cases by the end of 2014.^2^ The rapid spread of the virus overwhelmed the country’s health system. Due to the high caseload, the EVD surveillance system (consisting of case investigation of reported cases, contact tracing, screening of patients at health facilities and swab testing of corpses) could not rapidly identify and respond to alerts resulting in a high proportion of cases detected after death.^3^ As is usual for surveillance systems, there was no community level system to quickly detect and report new cases. In the absence of such system, the surveillance system was mainly passive, relying on the identification of suspect cases at facilities and contact tracing. This fueled the exponential spread of EVD across Sierra Leone.

The complexity of the response required coordinated support at the district level throughout the nation. The International Rescue Committee (IRC) initiated the creation of the Ebola Response Consortium (ERC) in August 2014 to support the Sierra Leone Ministry of Health and Sanitation (MoHS) in the EVD response. The ERC is a consortium of 15 NGOs with operational presence across the country backing this response through the implementation of district-wide programs in surveillance, infection prevention and control in primary health care facilities, and water, sanitation, and hygiene.

Although past EVD outbreaks highlighted the importance of community volunteers in detecting and reporting suspect cases, these outbreaks occurred across much smaller geographic contexts.^4^ Recognizing the opportunity to bring this concept to scale, the ERC partnered with the U.S. Centers for Disease Control and Prevention (CDC) and the MoHS to design and implement a community event-based surveillance (CEBS) system in 9 of the 14 districts of Sierra Leone beginning in January 2015. This was based on a pilot of the project in Bo district in October 2014.^3^ The objectives of CEBS were to improve the timeliness with which EVD cases were detected, isolated, and provided with the appropriate care before they created further chains of transmission. To fulfill the objective of early warning through increased sensitivity and rapid reporting from the village level, the ERC used a structured approach to identify events and rumors suggestive of EVD rather than case-based surveillance. Such event-based reporting is often used to detect new clusters of disease and to track health conditions at large events.^5^

This paper introduces the CEBS model, describes the ERC’s experiences during the first six months of CEBS implementation, and highlights key programmatic challenges. The overall aim is to better inform the design and implementation of community surveillance systems for future outbreaks of epidemic-prone disease. In parallel, an epidemiological evaluation of the effectiveness and sensitivity of the detection of confirmed cases was previously outlined and the results have been published elsewhere.^3^^,^ 6

## Methods

**Context**

Near the peak of the EVD epidemic in West Africa in October 2014, the IRC, Sierra Leone’s Bo District Health Management Team, and the US CDC developed CEBS to promote the early warning of EVD clusters at the village-level^3^ . National surveillance was not detecting infected persons until after they had died. The system comprised contact tracing, healthcare facility surveillance, and a telephone hotline for reporting events. As a result, opportunities for virus transmission in the community were prolonged^3^. CEBS was designed to supplement the surveillance system by training community members to identify unsafe burials and persons with signs and symptoms compatible with EVD infection. This made is possible to detect EVD cases that were not epidemiologically linked to other confirmed cases at the time of detection. In turn, this could provide early warning of unknown and new chains of transmission. The system was based primarily of a pre-existing network of community health workers in most of the districts.

**CEBS Model and Implementation**

At the base of the CEBS model are the Community Health Monitors, who are volunteers located in each community. Community Health Monitors are trained to detect and immediately report on a set of six trigger events that may be associated with EVD transmission (**Table 1**). Upon detecting a trigger event, the Community Health Monitors uses a mobile phone to inform his or her Community Surveillance Supervisor. The Community Surveillance Supervisor determines if an investigation is needed to assess whether a person meets criteria for a suspected EVD case. With the local Community Health Officer, a clinically-trained MoHS staff member working at the Chiefdom level, the Community Surveillance Supervisor conducts a preliminary screening of the alert. If the Community Surveillance Supervisor and Community Health Officer determine that a suspected case has occurred, they call in the alert to the District Ebola Response Center (DERC)—the emergency response unit set up by the national government to respond to all alerts—which dispatches a team to conduct a formal case investigation. A flow diagram of the system is presented in Figure 1.

**Flow diagram of CEBS:**
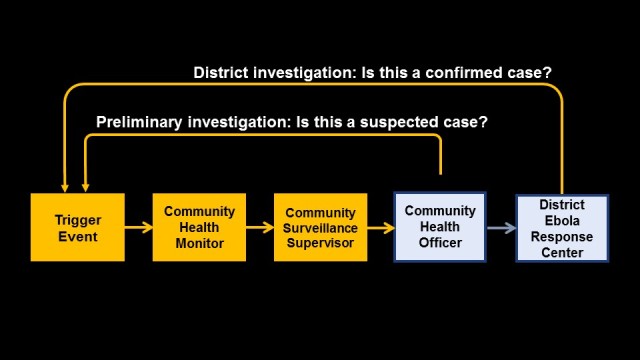

**Table 1:** **Table 1:** Trigger Events Detected by Community Health Monitors

	Trigger Events
1	Two or more family or household members become sick or die within a short period of time (less than seven days)
2	Anyone becomes sick or dies within three weeks of taking part in an unsafe burial or washing/touching a corpse
3	Any healthcare worker or traditional healer becomes sick or dies of an unknown cause
4	Any traveler (or recently returned traveler who is from that village) becomes sick or dies
5	Anyone who was a contact of a suspect EVD case (whether or not they were being contact traced) becomes sick or dies
6	Any unsafe burial or washing of a dead body that took place in the village or surrounding community (this trigger event would alert the surveillance and response team that there might be cases in the near future)

The CEBS model was designed and piloted in 100 villages in Bo district by the IRC, Bo District Health Management Team, and CDC during November 2014 as a complement to the passive EVD surveillance activities ongoing at the time.^3^ A consultative process involving participatory meetings with the Community Health Officers and Bo District Health Management Team was used to develop and refine the list of six trigger events to be detected by Community Health Monitors. The pilot findings were shared with the national MoHS in December 2014, who worked with IRC and CDC staff to develop a CEBS standard operating procedure to enable the ERC’s support of implementation at the national level.

In January 2015, ERC partners began supporting the implementation of CEBS in 9 of the 14 districts of Sierra Leone. To ensure standardized implementation across districts, all ERC partners adhered to the same six-step implementation plan included in the CEBS standard operating procedures (**Table 2**). This plan included creating district CEBS management teams (comprised of representatives of the ERC partner organization, the District Health Management Team, and the DERC), training Community Health Monitors and Community Surveillance Supervisors, procuring motorbikes and mobile phones, and setting up Closed User Group phone networks in each district with the goal of having all nine districts fully operational by the first week of March 2015. Closed User Group networks enable calling at no costs to all persons in the network.

**Table 2:** **Table** **2:** Key Steps to CEBS Implementation

	Step
1	Form a district CEBS management team consisting of representatives from the District Health Management Team, ERC partner, and other surveillance partners (e.g. CDC, WHO).
2	Introduce CEBS to district stakeholders and secure endorsement.
3	Introduce CEBS to Chiefdom stakeholders, including traditional leaders, and secure endorsement.
4	Identify and train Community Surveillance Supervisors and Community Health Officers in each Chiefdom.
5	Identify Community Health Monitors in each village in collaboration with traditional leaders. Community Health Officers and Community Surveillance Supervisors train the Community Health Monitors with support of district CEBS management team.
6	Establish a small team at the district level to plan and oversee CEBS data collection, analysis, and reporting.

The CEBS SOP recommended that one Community Health Monitor be selected per 50 households, while taking into consideration the geographic size and population density of each community. This ratio was based on the ratio used in the national Community Health Worker program.^7^ Similarly, at least one Community Surveillance Supervisor was selected per Chiefdom (sub-district level), depending on Chiefdom size, population density, and number of Community Health Monitors. The selection criteria for Community Health Monitors were that they should be respected residents of their communities with previous experience in a role of responsibility within their communities (such as teachers), while ideal candidates for the role of Community Surveillance Supervisor would be individuals with some health-related work experience who are capable of supervising others and had a strong knowledge of the Chiefdoms they serve.

Sierra Leone has had a community health worker program since 2006, and thus there was already a network of community health workers who had been elected by their communities and were already providing health promotion and basic health services before the EVD outbreak. Whenever possible, the community health workers and their supervisors were selected to serve as Community Health Monitors and Community Surveillance Supervisors, as it was believed that community health workers had already built the level of rapport and trust within their communities that would be critical for the successful functioning of CEBS.

**Alert system, Data compilation, and Analysis**

Community Health Monitors immediately notified their Community Surveillance Supervisors of detected triggers by mobile phone. This initiated the investigation process involving the Community Surveillance Supervisors and Community Health Officers. Each Community Surveillance Supervisor completed a weekly CEBS Alert Log where each alert raised by their Community Health Monitors are recorded along with the resulting response actions. The data captured on this form include the date, time, trigger event, the type of each alert (classified as sickness, death, unsafe burial, or “other”), as well as the name, age, sex, and location of the individual(s) being reported as sick or deceased. The Alert Log also contains sections to document suspicious events detected by the Community Health Monitors that were not represented by one of the triggers (classified as “Trigger 7- Other”). The Community Surveillance Supervisors update these forms every time an alert is received and submit their forms to the district CEBS team at the end of each week. On a weekly basis, the team entered the data into an Excel-based reporting tool and submits it to the ERC coordinating unit for cleaning and compilation into a central database. For this evaluation, the central CEBS database was used to analyze the alerts generated by CEBS between March and August 2015. Descriptive analyses were conducted by disaggregating the total number of alerts by alert trigger event and type.

Each Community Surveillance Supervisor also keeps a Community Health Monitor Weekly Reporting Form where they track how often each Community Health Monitor reports to them and how many alerts they report. Each Community Health Monitor is expected to report to their Community Surveillance Supervisor at least once per week, even if they have not detected any alerts. This “zero reporting” feature indicates that the Community Health Monitor is active and looking for triggers in their community. Zero reporting was only feasible on a weekly basis as a double check. Daily zero reporting would have been overwhelming for Community Surveillance Supervisors. On the other hand, event reporting was immediate. This form is also submitted to the district CEBS team at the end of each week and a summary is submitted weekly to the ERC coordinating unit.

**Process Evaluation of CEBS**

In this evaluation, the routine data were used to analyze: (1) the proportion of Community Health Monitors reporting at least once per week and (2) the number of alerts reported by Community Health Monitors between March and August 2015 disaggregated by type and trigger. Between April and June 2015, the ERC, IRC, and CDC conducted a process evaluation of CEBS in the nine districts to evaluate how acceptable the CEBS structure was to Community Health Monitors, Community Surveillance Supervisors and other partners on the ground, in addition to knowledge and attitudes of Community Health Monitors and Community Surveillance Supervisors. The assessment included key informant interviews with approximately 50 Community Health Monitors, 27 Community Surveillance Supervisors, and 31 district stakeholders (such as District Health Management Teams, DERCs, and WHO). A semi-structured questionnaire was used to guide the interviews, which included quantifiable responses and open-ended questions. Due to the rapid nature of the assessment, Community Health Monitors and Community Surveillance Supervisors were selected using a purposive sample. We aimed to include at least two Chiefdoms that were close to, and far away from, the district capital in order to assess a variety of contexts. Quantitative interview data were aggregated and qualitative data were compiled for analysis.

**** **Ethical review**

This assessment was a part of a nonresearch public health response activity and thus did not undergo institutional review board review. In addition, we used only information that had already been collected for public health surveillance purposes, so informed consent was not obtained.

## Results

**Implementation Process**

Implementation began in January 2015 with the formation of the district CEBS teams, completion of the district- and Chiefdom-level stakeholder meetings, and implementation planning (comprising steps 1 to 3 in Table 2). The purpose of the district- and Chiefdom-level stakeholder meetings was to secure endorsement from local leaders and encourage community ownership and participation in the program. The Community Health Monitor trainings began in mid-February and were completed in all nine ERC districts by the end of March. While the stakeholder meetings and trainings were ongoing, ERC partners began the process of procuring motorbikes and mobile phones and creating Closed User Groups to allow Community Surveillance Supervisors and Community Health Monitors to call each other free of charge. The process of working with telecommunication companies to establish large Closed User Groups was time-consuming. By August 2015, Closed User Groups had not been established in three of the nine districts. In place of Closed User Groups, Community Health Monitors and Community Surveillance Supervisors in these districts were provided with a monthly allowance of pre-paid phone credit. Delays were also experienced with the procurement, licensing, and registration of motorbikes. Motorbikes were fully procured and licensed in all nine operational districts by July 2015.

**CEBS Coverage**

A total of 137 Community Surveillance Supervisors and 7,142 Community Health Monitors were trained across the nine districts to cover an estimated population of 3,981,665 (approximately 63% of the total projected population for Sierra Leone).^8^ Across the nine districts, the average number of households overseen by a Community Health Monitor was 118 to 1, while the ratio of Community Health Monitors to Community Surveillance Supervisors was 52 to 1. The household coverage varied by district and ranged from 78 households per Community Health Monitor (in Kono and Kambia) to 184 households per Community Health Monitor (in Bombali). Similar variations exist in the number of Community Health Monitors per Community Surveillance Supervisors, which ranges from 24 Community Health Monitors per Community Surveillance Supervisor in Moyamba to 68 Community Health Monitors per Community Surveillance Supervisor in Bo and Kambia (**Table 3**).

**Table 3:** **Table 3**: CEBS Coverage in ERC Districts

District	Estimated Population	Households	CSSs Trained	CHMs Trained	CHM:CSS Ratio	Household:CHM Ratio
Bo	654,142	131,396	18	1228	68:1	107:1
Bombali	494,139	100,832	15	548	36:1	184:1
Kailahun	465,048	93,288	14	676	48:1	138:1
Kambia	341,690	68,640	13	880	68:1	78:1
Kenema	653,013	130,779	20	1321	66:1	99:1
Kono	325,003	65,130	15	835	56:1	78:1
Moyamba	278,119	55,752	17	404	24:1	138:1
Pujehun	335,574	67,000	12	500	42:1	134:1
Tonkolili	434,937	87,000	13	750	58:1	116:1
Total or average	3,981,665	842,756	137	7,142	52:1	118:1

CSS: Community Surveillance Supervisor; CHM: Community Health Monitor

**Community Health Monitor Reporting and CEBS Alerts**

Community Health Monitors in three districts (Moyamba, Pujehun, and Tonkolili) began reporting in March, five districts (Bo, Bombali, Kambia, Kenema, and Kono) began in April and May, and Community Health Monitors in Kailahun began reporting in June. Every district experienced an initial lag in reporting in their first month of operation, with increasing reporting coverage in the following months. The number of alerts reported by Community Health Monitors increase from 401 in March, when three districts were fully operational, to 1849 in June when all districts were operational. For the following two months, the number of alerts continued to increase to 2199 in July and 2330 in August (**Figure 2**).

**Number of CEBS Alerts by District and by Month, March-August 2015:**
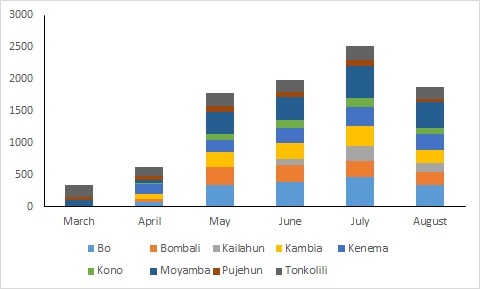

The average proportion of Community Health Monitors reporting alerts or the absence of alerts at least once per week increased from March to May, decreased in June, and increased again through July and August. The June decrease can be attributed to the low percentage of Community Health Monitors reporting in Kailahun, the first month of Community Health Monitors reporting for that district. In August, 92% of Community Health Monitors across the nine districts reported alerts or a zero report at least once per week (**Table 4**).

**Table 4:** **Table 4: **Proportion of Community Health Monitors Reporting at least Once per Week, March-August 2015

District	March	April	May	June	July	August	Average
Bo	-	74%	92%	96%	91%	93%	89%
Bombali	-	80%	93%	98%	98%	98%	93%
Kailahun	-	-	-	28%	74%	85%	62%
Kambia	-	64%	82%	96%	94%	95%	86%
Kenema	-	54%	97%	92%	96%	95%	87%
Kono	-	-	87%	95%	95%	97%	93%
Moyamba	23%	63%	97%	98%	94%	96%	78%
Pujehun	22%	53%	65%	52%	74%	73%	56%
Tonkolili	70%	93%	97%	92%	95%	93%	90%
Average	38%	69%	89%	83%	90%	92%	82%

Of the 9,131 alerts generated in the period under review, 7,930 alerts (87%) represented deaths, while 1,183 (13%) represented illnesses (**Table 5**). 8,627 (94%) were reported as “Trigger 7- Other” meaning that the Community Health Monitor did not classified them as one of the six trigger events that Community Health Monitors were trained to detect. Among the 9,131 alerts, the most commonly reported of the six trigger events were “two or more sick/dead in same household” (n=194, 2.1%) and “sick/death among traveler” (n=158, 1.7%).

**Table 5:** **Table 5: **CEBS Alerts by Trigger Event and Alert Type, March-August 2015

Trigger Event	Death	Other	Sick	Unsafe burial	Total
Two or more sick/dead in same household	132	0	62	0	194
Sick/death after unsafe burial/corpse washing	54	0	5	0	59
Sick/death among health worker/healer	40	0	18	0	58
Sick/death among traveler	100	0	58	0	158
Sick/death in contact of EVD case	16	0	12	0	28
Unsafe burial/corpse washing	6	0	0	1	7
Other	7582	17	1028	0	8627
Total	7930	17	1183	1	9131

**Note:** Five of the six alerts identified through the unsafe burials trigger were classified as deaths under “alert type”. It is unclear whether these alerts were actually for unsafe burials (in which case the alert type would be categorized incorrectly) or for deaths occurring after an unsafe burial (in which case the trigger event would be categorized incorrectly). Given this uncertainty and the impossibility to recode either alert type or trigger event with 100% certainty, no changes have been made to the data.

**Community Health Monitors and Community Surveillance Supervisor Interviews**

The rapid assessment of CEBS involved interviews with 50 Community Health Monitors, 27 Community Surveillance Supervisors and 31 stakeholders. We found that Community Health Monitors recalled, on average, three of the six trigger events. Twenty of 50 Community Health Monitors (40%) remembered between 1 and 3 triggers, while 24 Community Health Monitors (48%) remembered between 4 and 6 trigger events. As shown in **Figure 3**, some triggers were more frequently recalled than others. The most commonly-recalled triggers were those concerning an illness or death among a traveler (35/50, 70%) and an illness or death among two or more members of the same household (33/50, 66%). The least frequently-recalled trigger was the occurrence of illness or death among a contact of an EVD case, which was only recalled by 14 (28%) Community Health Monitors.

All Community Health Monitors reported that they actively seek information about illnesses and deaths in their communities. Strategies mentioned by Community Health Monitors included visiting households, speaking to community leaders, or speaking with other key informants such as teachers and health care workers. Sixty-eight percent reported that their community supports their work. The 27 Community Surveillance Supervisors interviewed recalled an average of five of the seven actions they were trained to take when they receive an alert from a Community Health Monitors. The most common challenges Community Health Monitors and Community Surveillance Supervisors reported was the malfunction of the Closed User Group phone system and lack of motorbike.

**Proportion of Community Health Monitors Who Recalled Each Trigger Event:**
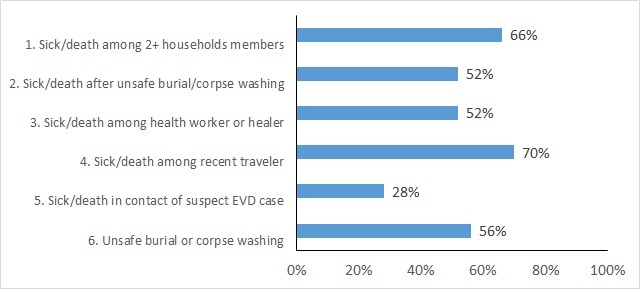

**Interviews with District Stakeholders**

Stakeholders interviewed included representatives from the District Health Management Team, such as District Medical Officers and District Surveillance Officers, as well as members of other district EVD response agencies, such as DERC Coordinators, WHO Field Coordinators, and CDC epidemiologists. Eighteen stakeholders (58%) had a general understanding of the structure and function of CEBS. Twenty-three stakeholders (74%) interviewed stated that CEBS had benefited their district through enhanced community engagement and/or increased detection of suspected EVD cases. Stakeholders discussed challenges and concerns related to CEBS. The need for strengthened coordination between CEBS and other EVD response activities in terms of the sharing of surveillance reports was the most frequently-cited challenge and was noted by 13 (42%) interviewees. The sustainability of CEBS—both financially and programmatically when the DERCs begin to scale down—was also a major concern that was noted in eight (26%) of the interviews.

## Discussion

Few surveillance systems in humanitarian emergencies are formally described and evaluated to assess efficiency and effectiveness^9^^,^ 10. This report attempts to assess the efficiency and implementation strategies of a national, disease-specific surveillance system. The rapid implementation of CEBS at a national scale in Sierra Leone was estimated to take two months to become fully operational by March 2015. In practice, the implementation process took six months and all nine districts began reporting alerts in June 2015, well after the peak in caseload. Although the planning and stakeholder meetings were time-consuming, the findings of the field assessment interviews indicate that the program was well-received by Community Health Monitors, Community Surveillance Supervisors and community members. This community ownership was an important aspect of CEBS considering the sensitive nature in some communities of reporting EVD cases and events during the outbreak.

We note three main advantages of a community based surveillance system. First, monthly reporting is considered to be very high (>80%) for most months in operation. The first six months of implementation saw a steady increase in the percentage of Community Health Monitors reporting at least once each week and the number of alerts reported by Community Health Monitors. In August 2015, 92% of Community Health Monitors reported at least once each week to their Community Surveillance Supervisors, indicating that most Community Health Monitors were actively performing community surveillance and are able to communicate with their Community Surveillance Supervisors. Second, reporting was consistent across time and geography. CEBS generated consistent surveillance information during periods of no- to low- transmission across districts when contact tracing was no longer active. This provided additional information to use to rule-out the presence of transmission during quiet periods. Third, the introduction of a community–based element to surveillance made a sufficient and satisfying linkage between communities and the overall EVD response. This was indicated by the satisfaction level of community members that was reported by Community Health Monitors.

Despite these advantages, there were three important weaknesses of the system. First, logistical delays proved to be the biggest challenge for CEBS implementation and resulted a delay in achieving geographical coverage goals until most districts were no longer documenting EVD cases. Issues with large-scale procurement of vehicles and the Closed User Group resulted in trained Community Health Monitors and Community Surveillance Supervisors who were unable to carry out their responsibilities effectively. The lack of communication and transportation may be responsible for the low number of alerts reported in the first three months of implementation. In a future crisis, it is advisable to search for preexisting sources transportation means and streamline the setup of Closed User Groups or distribution of mobile credit through a central source rather than a district-based approach in order to speed up the implementation process or prioritize procurement as a first step in implementation.

Second, the anticipated ratio of Community Health Monitors to population and Community Health Monitors to Community Surveillance Supervisors between districts was not consistently achieved. Partners were given flexibility within the SOP to establish the network of Community Health Monitors and Community Surveillance Supervisors themselves and took different approaches to doing so, with some districts using only pre-existing community health workers while others selected additional Community Health Monitors in areas that were not previously covered by a community health worker. To get the system running as quickly as possible, it was decided that some decisions would be decentralized and made at the district level, instead of the national level. Though the CEBS SOP recommended a ratio of one Community Health Monitor per 50 households, a ratio of one Community Health Monitor per 118 households was achieved across the nine districts. It is not known how much this higher ratio affected the Community Health Monitors’ ability to detect trigger events in their communities.

****Third, the distribution of trigger events favored deaths over live alerts. Eighty-seven percent of alerts reported by Community Health Monitors were for deaths, most of which were not a trigger event and were not alerted as deaths linked to EVD. Since early on in the EVD outbreak, the MoHS has required that all deaths, regardless of cause, be reported to DERCs in order for the body to receive a swab test for EVD and be buried safely by a trained burial team. Nationally, the requirement for reporting deaths has been widely and frequently communicated to the population. CEBS was not intended to serve as a reporting system for community deaths, but assumed this role as many Community Health Monitors either felt it was their responsibility to report all deaths or were required to by policies pertaining to safe burial. In many respects, this was a positive development, as community death reporting is a means to both monitor whether Community Health Monitors are staying active and the activity helped to confirm zero transmission in districts without transmission. However, our data cannot describe the validity or completeness of this death reporting. Clustering of illnesses and deaths (ie, trigger 1) was reported less frequently than anticipated. The low proportion of reporting sick alerts can be partially attributed to lack of clarity about who is considered “sick.” Due to the prevalence of disease in Sierra Leone, illness is a daily occurrence in rural communities of Sierra Leone. Community Health Monitors may not consider a person to be sick unless they have severe or unusual symptoms. More refinement of this trigger is needed, as such indications of clustering are the major link to early warning of transmission at the community level. It is informative that during the rapid evaluation, most Community Health Monitors interviewed did not recall all six triggers. The challenges in trigger recall may be due, in part, to the lack of EVD transmission by the time CEBS became fully operational, resulting in a lack of Community Health Monitor exposure to these events, which were much more common during peak transmission during late 2014 and early 2015. This challenge may also indicate that there are too many triggers for Community Health Monitors to easily remember and the content may be too abstract. Notably, the most commonly-reported of the six trigger events, including illness or death among a traveler and an illness or death among two or more members of the same household, were also the most commonly reported triggers after “Trigger 7- Other”. These two events are likely the easiest for Community Health Monitors to understand and the easiest to detect.

There are important limitations to this evaluation. While the population-to-Community Health Monitor ratios presented are useful in giving a broader view of the overall distribution of Community Health Monitors, they are not able to truly assess CEBS coverage through identifying which villages have Community Health Monitors and which villages do not. In addition, population ratios are based off of data from the 2004 census that have been extrapolated to 2014 based on expected annual population increases, which does not take into account migration between and within districts. The Community Health Monitors and Community Surveillance Supervisors interviewed during the field assessment were selected via purposive sampling due to time and logistical constraints that would have been posed by random sampling. However, we aimed for representation geographically within each district. In addition, the sampling fractions were small, with interviews being conducted with only 50 of the 7,050 Community Health Monitors (0.7%) and 27 of the 137 Community Surveillance Supervisors (20%).

## Conclusion

The implementation of CEBS has shown that a national system can be implemented at scale and that community volunteers are capable of detecting and reporting important health related events in their communities. Participation of community leaders in the implementation process proved to be an important step in ensuring that communities are supportive of the program and Community Health Monitors are able to carry out their responsibilities effectively. The experience with CEBS implementation shows that planning to implement a large-scale community based program in two months across many partners requires well-planned and well-coordinated logistic procedures. The system as it stands may be utilized to inform the long-term Integrated Disease Surveillance and Response system.

## Competing Interests

The authors have declared that no competing interests exist.

## Data Availability

Data is available upon request. Please contact Laura Miller, laura.miller@rescue.org.
